# Correction: A novel human *IL2RB* mutation results in T and NK cell–driven immune dysregulation

**DOI:** 10.1084/jem.2018201505102019c

**Published:** 2019-05-13

**Authors:** Isabel Z. Fernandez, Ryan M. Baxter, Josselyn E. Garcia-Perez, Elena Vendrame, Thanmayi Ranganath, Daniel S. Kong, Karl Lundquist, Tom Nguyen, Sidney Ogolla, Jennifer Black, Csaba Galambos, James C. Gumbart, Noor Dawany, Judith R. Kelsen, Edwin F. de Zoeten, Ralph Quinones, Hesham Eissa, Michael R. Verneris, Kathleen E. Sullivan, Rosemary Rochford, Catherine A. Blish, Ross M. Kedl, Cullen M. Dutmer, Elena W.Y. Hsieh

Vol. 216, No. 6, June 3, 2019. 10.1084/jem.20182015.

The authors regret that in the original version of Fig. S2 G, the plot for healthy pediatric (H. Ped) #1 CD4^+^ T cells was mistakenly replicated in the healthy adult (H. Adult) plot. The corrected panel appears below.

**Figure:**
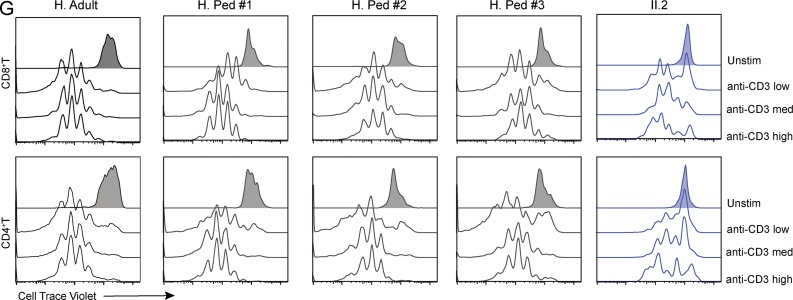


The online supplemental PDF has been corrected. The error appears only in supplemental PDFs downloaded before May 9, 2019.

